# Associations between weight-adjusted waist index and bone mineral density: results of a nationwide survey

**DOI:** 10.1186/s12902-023-01418-y

**Published:** 2023-08-03

**Authors:** Ya Zhang, Haiyang Wu, Cheng Li, Changxiong Liu, Mingjiang Liu, Xiaozhu Liu, Qiming Yin, Xianzhe Li, Ruijie Xie

**Affiliations:** 1 Department of Gland Surgery, The Affiliated Nanhua Hospital, Hengyang Medical school, University of South China, Hengyang, China; 2 Duke Molecular Physiology Institute, Duke University School of Medicine, Duke University, Durham, NC USA; 3 Department of Orthopaedic Surgery, Beijing Jishuitan Hospital, Fourth Clinical College of Peking University, Beijing, China; 4 Department of Microsurgery, The Affiliated Nanhua Hospital, Hengyang Medical school, University of South China, Hengyang, China; 5 Department of Cardiology, The Second Affiliated Hospital of Chongqing Medical University, Chongqing, China; 6 Division of Clinical Epidemiology and Aging Research, German Cancer Research Center (DKFZ), Heidelberg, Germany

**Keywords:** Weight-adjusted-waist, Bone mineral density, Osteoporosis, NHANES, Obesity

## Abstract

**Background:**

The weight-adjusted waist circumference index (WWI) is a novel obesity indicator that offers improved accuracy in assessing both muscle and fat mass compared to traditional measures. This study aimed to investigate the association between WWI and bone mineral density (BMD) in adults.

**Methods:**

Weighted multivariate logistic regression, subgroup analysis, interaction tests and restricted cubic spline (RCS) curves were used to explore the relationship between WWI and BMD based on data from the National Health and Nutrition Examination Survey (NHANES).

**Results:**

This study had 40,568 individuals in total. At all four measurement sites, we detected a negative linear correlation between WWI and BMD. Even when quartile factors for WWI were created, this unfavorable connection maintained. In comparison to those in the lowest quartile, those in the highest percentile of WWI showed declines in lumbar BMD of 0.08 g/cm^2^ and femoral neck BMD of 0.03 g/cm^2^, respectively. This adverse correlation, nevertheless, differed among several categories.

**Conclusions:**

Our findings suggest an adverse correlation between WWI and BMD among US adults. Employing WWI as a tool for osteoporosis prevention in the general population may enhance interventions.

**Supplementary Information:**

The online version contains supplementary material available at 10.1186/s12902-023-01418-y.

## Background

Osteoporosis is a pervasive public health problem characterized by low Bone Mineral Density (BMD), making early prevention and risk factor identification crucial [1, 2]. Population-based research advances have resulted in more accurate evaluations of fracture risk and a broader choice of fracture prevention methods [3, 4].

Obesity is a complicated metabolic disorder [5]. Obesity prevalence has increased significantly globally, with roughly 30% of the world’s individuals currently afflicted [6, 7]. Traditional obesity markers, such as BMI and waist circumference (WC), have limitations, notably in their capacity to distinguish between muscle and fat mass, which may lead to less accurate estimates of an individual’s health risk [8–10]. As a result, it has been suggested that body composition and body fat distribution more closely reflect negative metabolic features [11, 12].

In contrast, the weight-adjusted waist circumference index (WWI) has emerged as a potentially more reliable and informative obesity indicator [13, 14]. It has been associated with age-related changes in body composition and has been linked to various health conditions, suggesting its potential as a valuable tool in evaluating health risks [15–19].

Despite the promise that WWI holds as an obesity indicator, the relationship between WWI and bone metabolism has not been previously explored in the scientific literature. In response to this knowledge gap, the present study aims to explore the association between WWI and BMD by analyzing data from the National Health and Nutrition Examination Survey (NHANES), which was conducted between 1999 and 2018.

## Methods

### Study participants

**Fig. 1:**
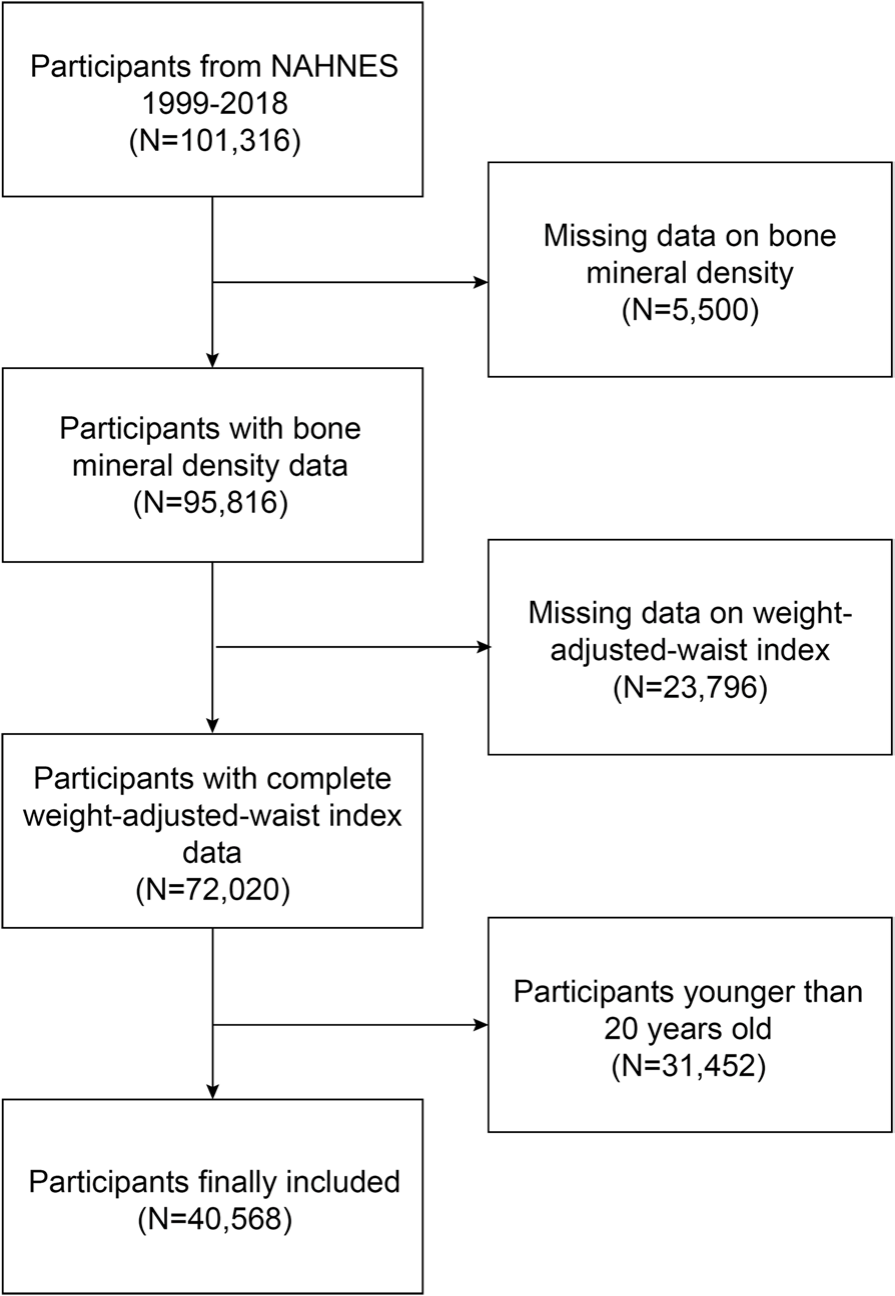
Flow chart of participants selection. NHANES, National Health and Nutrition Examination Survey

The National Center for Health Statistics (NCHS) conducts the well-known National Health and Nutrition Examination study (NHANES), a cross-sectional study that is nationally representative [20–22]. All research participants provided written agreement at the time of recruitment, and the NCHS Research Ethics Review Board approved the study’s methodology. Over 10 survey cycles in a period of twenty years (1999–2018), the survey was carried out. We removed 31,452 people under the age of 20, 23,796 participants with insufficient WWI data, and 5,500 participants without relevant BMD data. The final number of participants in the research was 40,568 (Fig. 1).

### WWI

The WWI is determined by dividing waist circumference (cm) by the square root of body weight (kg), is a tool for assessing body fat mass and muscle mass [23]. Certified health professionals took participants’ weights and waist circumferences at the mobile examination facility. By taking off their shoes and bulky clothing, the participants’ weights were calculated. The waist circumference was estimated by drawing a horizontal line above the highest lateral border of the right iliac bone and then inserting a tape measure at the junction of the two lines [24].

### BMD

BMD values were evaluated using a dual-energy X-ray absorptiometry scan in four separate areas, including the lumbar, pelvic, femoral neck, and total BMD, as in prior research, to reduce bias across diverse populations [25]. Supplementary file 1 details from which file the BMD of the different parts was extracted.

### Covariables

Covariates included age, sex, smoking status, dietary inflammatory index, low-density lipoprotein cholesterol (LDL-C), race, cancer, high blood pressure, use of hormone medication, take prescription for cholesterol, diabetes, income-to-poverty ratio (PIR), triglycerides, education level, and history of bone fracture.

### Statistical analysis

Considering that NHANES uses a complex multi-stage probability sampling design. To assess participant demographics by WWI quartile, we employed the chi-square test and t-test. For examining the linear relationship between WWI and BMD, we used weighted multivariate logistic regression analysis. After categorizing WWI into quartiles, we utilized a trend test to analyze the linear association trend between WWI and BMD. Additionally, subgroup analysis was carried out to investigate the relationship between WWI and BMD in various subpopulations based on factors such as sex, race, education, high blood pressure, and diabetes status. We also conducted interaction tests to assess the consistency of the associations across subgroups. To investigate the nonlinear association between WWI and BMD, we used restricted cubic spline (RCS) curve. We established statistical significance at two-sided *P* < 0.05 [26, 27]. The statistical software packages used were R (version 4.2), Python (version 3.10.4) and Empowerstats (version 5.0).

## Results

### Baseline characteristics

The study included 40,568 participants, with a mean (SD) age of 48.74 (18.01) years, and 51.56% of participants were female. The mean (SD) BMI and WWI for all participants were 28.49 (6.31) kg/m^2^ and 11.01 (0.85) cm/√kg. Compared with participants in the lowest WWI quartile, those in the highest quartile were more likely to be female, Mexican American, and elderly. Participants with higher WWI had lower education and income levels, higher smoking rates, higher use of hormone and lower cholesterol medication, greater dietary inflammatory potential, a greater history of diabetes, high blood pressure, cancer, and fractures, as well as higher cholesterol levels and lower BMD (Table 1).

**Table 1:** Basic characteristics of participants by weight-adjusted-waist index quartile

Characteristics	Weight-adjusted-waist index				*P*-value
	Q1 (< 9.84) *N* = 1,731	Q2 (9.84–10.55) *N* = 1,730	Q3 (10.56–11.27) *N* = 1,731	Q4 (> 11.27) *N* = 1,731	
Age (years)	36.86 ± 13.10	44.71 ± 14.65	50.96 ± 15.84	57.20 ± 16.88	< 0.001
Sex, (%)					< 0.001
Male	55.42	53.08	46.60	33.48	
Female	44.58	46.92	53.40	66.52	
Race/ethnicity, (%)					< 0.001
Non-Hispanic White	69.64	68.82	67.90	68.86	
Non-Hispanic Black	14.03	9.49	9.32	8.58	
Mexican American	4.91	8.66	10.27	10.40	
Other race/multiracial	11.41	13.03	12.50	12.16	
Education level, n (%)					< 0.001
Less than high school	12.04	15.39	20.37	25.83	
High school	20.21	23.48	24.90	26.10	
More than high school	67.75	61.13	54.73	48.07	
Smoking, (%)					< 0.001
Ever	41.99	46.44	48.90	50.10	
Never	58.01	53.56	51.10	49.90	
Diabetes, (%)					< 0.001
Yes	1.83	4.64	9.29	20.05	
No	98.17	95.36	90.71	79.95	
Cancer, (%)					< 0.001
Yes	4.20	6.98	10.49	13.52	
No	95.80	93.02	89.51	86.48	
High blood pressure, (%)					< 0.001
Yes	15.44	29.50	40.34	53.91	
No	84.56	70.50	59.66	46.09	
Take prescription for cholesterol, (%)					< 0.001
Yes	1.53	4.60	8.86	13.59	
No	98.47	95.40	91.16	86.41	
Hip fractured					< 0.001
Yes	0.54	0.89	1.19	1.26	
No	99.46	99.11	98.81	98.74	
Wrist fractured					0.027
Yes	11.10	9.22	10.15	10.37	
No	88.90	90.78	89.85	89.63	
Spine fractured					< 0.001
Yes	1.78	1.77	2.57	2.71	
No	98.22	98.23	97.43	97.29	
Use of hormone medication, (%)					< 0.001
Yes	1.45	2.58	2.85	3.99	
No	98.55	97.42	97.15	96.01	
BMI (kg/m^2^)	24.56 ± 4.35	27.66 ± 5.01	29.79 ± 5.66	32.85 ± 7.18	< 0.001
Waist circumference (cm)	84.65 ± 10.07	95.45 ± 11.00	102.72 ± 12.00	112.42 ± 15.07	< 0.001
PIR	3.18 ± 1.65	3.15 ± 1.63	2.98 ± 1.64	2.57 ± 1.58	< 0.001
DII	1.01 ± 1.86	1.27 ± 1.81	1.47 ± 1.78	1.74 ± 1.75	< 0.001
Triglycerides (mg./dL)	106.40 ± 92.04	131.69 ± 101.96	147.06 ± 138.60	157.97 ± 140.90	< 0.001
LDL-C (mg/dL)	110.34 ± 33.20	119.28 ± 34.51	120.29 ± 37.35	117.02 ± 36.57	< 0.001
Lumbar BMD (g/cm^2^)	1.07 ± 0.15	1.03 ± 0.14	1.01 ± 0.15	1.00 ± 0.16	< 0.001
Pelvis BMD (g/cm^2^)	1.28 ± 0.18	1.29 ± 0.17	1.27 ± 0.17	1.21 ± 0.17	< 0.001
Femoral neck BMD (g/cm^2^)	0.86 ± 0.15	0.83 ± 0.15	0.81 ± 0.14	0.78 ± 0.15	
Total BMD (g/cm^2^)	1.16 ± 0.11	1.14 ± 0.11	1.11 ± 0.11	1.08 ± 0.12	

Mean ± SD for continuous variables: the P value was calculated by the weighted linear regression model

(%) for categorical variables: the P value was calculated by the weighted chi-square test

*Abbreviation:Q *Quartile, *PIR *Ratio of family income to poverty, *BMI *Body mass index, *LDL-C *Low-density lipoprotein cholesterol, *DII *Dietary inflammatory index, *BMD *Bone mineral density

### Association between WWI and BMD

**Fig. 2:**
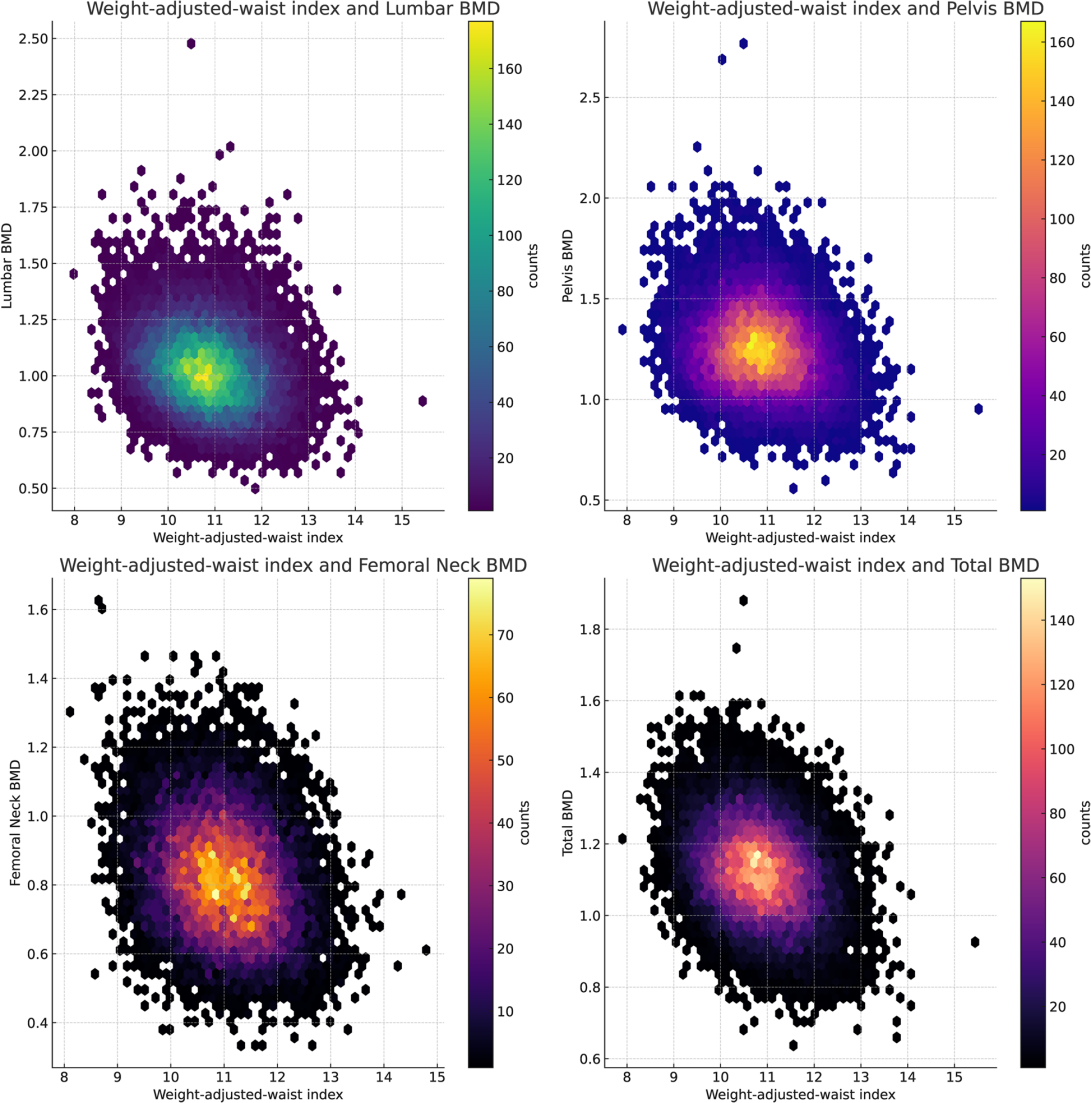
Hexbin Plots of weight-adjusted waist index and various bone mineral densities. Each subplot represents a different bone mineral density: Lumbar BMD (top left), Pelvis BMD (top right), Femoral Neck BMD (bottom left), and Total BMD (bottom right). The color of each hexbin represents the number of data points within that area, with darker colors indicating a higher count. Note that due to missing values in the dataset, not all potential data points are shown

**Fig. 3:**
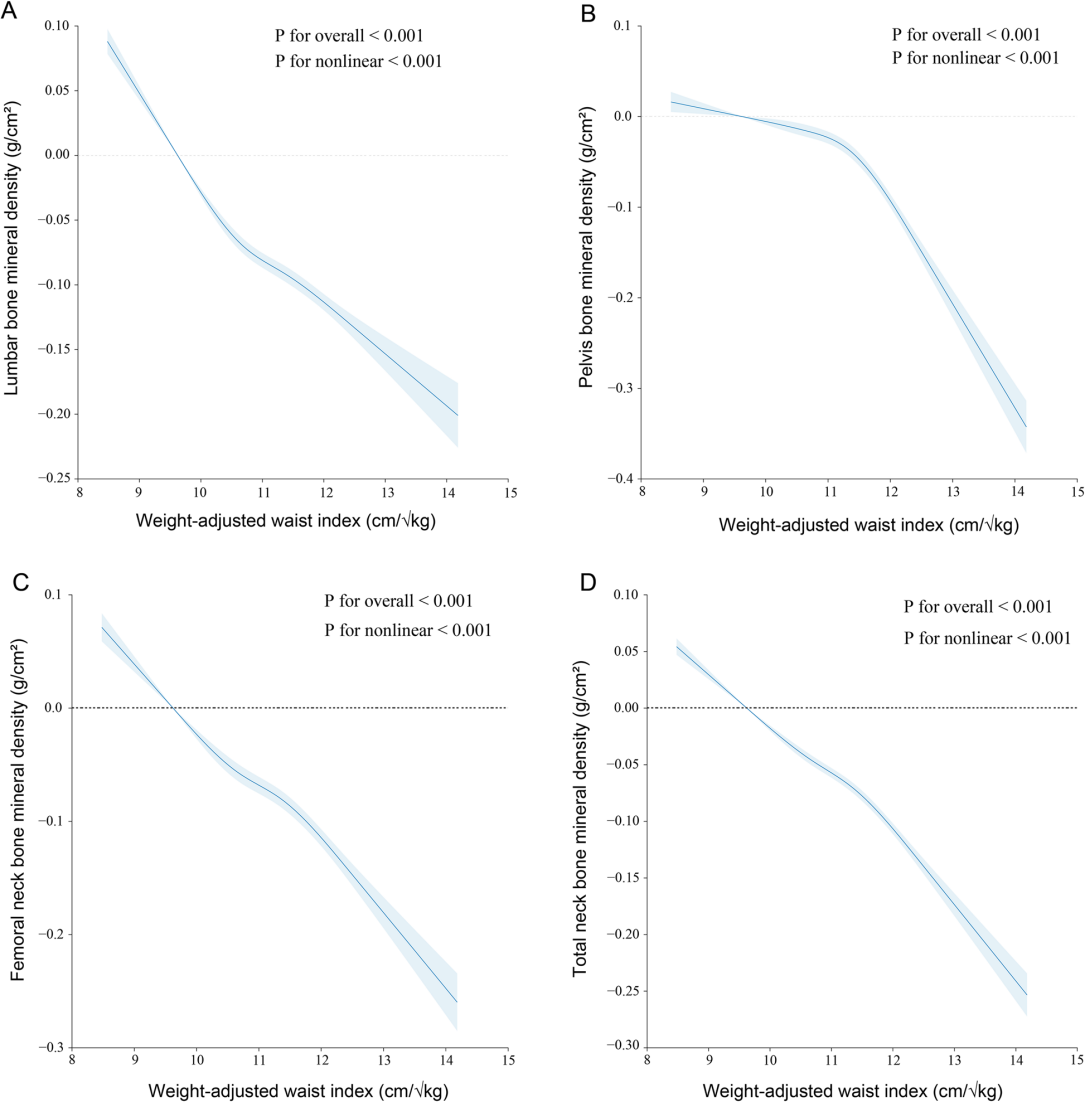
The nonlinear associations between weight-adjusted waist index and bone mineral density. The solid line represents the smooth curve fit between variables. Blue bands represent the 95% of confidence interval from the fit. **A** WWI and lumbar BMD; **B** WWI and pelvis BMD; **C** WWI and femoral neck BMD; **D** WWI and total BMD. WWI, weight-adjusted waist index; BMD, bone mineral density

The associations between WWI and BMD are presented in Table 2. In the crude and adjusted models, WWI was negatively correlated with BMD at all four sites. Thank you for your clarification. We appreciate your attention to detail and recognize the importance of conveying our findings clearly. In the fully adjusted model, each one-unit increase in WWI was associated with a decrease in BMD, measured in g/cm^2^, at all four sites. Specifically, we observed a decrease of 0.03 g/cm^2^ in lumbar BMD, 0.04 g/cm^2^ in pelvis BMD, 0.02 g/cm^2^ in femoral neck BMD, and 0.02 g/cm^2^ in total BMD. When comparing those in the highest quartile of WWI to those in the lowest quartile, we observed decreases in lumbar, pelvis, femoral neck, and total BMD by 0.08 g/cm^2^, 0.06 g/cm^2^, 0.03 g/cm^2^, and 0.05 g/cm^2^, respectively. Hexbin plots showing the relationship between the WWI and various bone mineral densities (Fig. 2). Smoothed curve fitting findings confirmed the nonlinear negative connection between WWI and BMD at all four sites (Fig. 3).

**Table 2:** The associations between weight-adjusted-waist index and bone mineral density

Exposure	Model 1 [β (95% CI)]	Model 2 [β (95% CI)]	Model 3 [β (95% CI)]
Lumbar BMD (continuous)	-0.04 (-0.04, -0.04)	-0.06 (-0.06, -0.05)	-0.03 (-0.04, -0.01)
Lumbar BMD (quartile)			
Quartile 1	reference	reference	reference
Quartile 2	-0.04 (-0.04, -0.03)	-0.05 (-0.05, -0.04)	-0.05 (-0.08, -0.02)
Quartile 3	-0.06 (-0.07, -0.05)	-0.07 (-0.08, -0.07)	-0.07 (-0.10, -0.04)
Quartile 4	-0.08 (-0.08, -0.07)	-0.10 (-0.11, -0.10)	-0.08 (-0.11, -0.04)
P for trend	< 0.001	< 0.001	< 0.001
Pelvis BMD (continuous)	-0.02 (-0.03, -0.02)	-0.03 (-0.04, -0.03)	-0.04 (-0.06, -0.03)
Pelvis BMD (quartile)			
Quartile 1	reference	reference	reference
Quartile 2	0.01 (0.01, 0.02)	0.01 (0.00, 0.01)	0.00 (-0.03, 0.04)
Quartile 3	-0.00 (-0.01, 0.00)	-0.02 (-0.02, -0.01)	-0.03 (-0.06, 0.01)
Quartile 4	-0.07 (-0.07, -0.06)	-0.08 (-0.09, -0.08)	-0.06 (-0.10, -0.03)
P for trend	< 0.001	< 0.001	< 0.001
Femoral neck BMD (continuous)	-0.04 (-0.04, -0.04)	-0.03 (-0.04, -0.03)	-0.02 (-0.03, -0.01)
Femoral neck BMD (quartile)			
Quartile 1	reference	reference	reference
Quartile 2	-0.03 (-0.03, -0.02)	-0.03 (-0.03, -0.02)	-0.00 (-0.04, 0.03)
Quartile 3	-0.05 (-0.05, -0.04)	-0.04 (-0.05, -0.03)	-0.02 (-0.04, 0.01)
Quartile 4	-0.08 (-0.09, -0.08)	-0.06 (-0.07, -0.06)	-0.03 (-0.05, -0.01)
P for trend	< 0.001	< 0.001	< 0.001
Total BMD (continuous)	-0.04 (-0.04, -0.04)	-0.04 (-0.05, -0.04)	-0.02 (-0.03, -0.01)
Total BMD (quartile)			
Quartile 1	reference	reference	reference
Quartile 2	-0.02 (-0.02, -0.02)	-0.02 (-0.03, -0.02)	-0.02 (-0.04, 0.00)
Quartile 3	-0.04 (-0.05, -0.04)	-0.05 (-0.05, -0.05)	-0.04 (-0.07, -0.02)
Quartile 4	-0.08 (-0.08, -0.08)	-0.09 (-0.09, -0.08)	-0.05 (-0.07, -0.02)
P for trend	< 0.001	< 0.001	< 0.001

Model 1: no covariates were adjusted. Model 2: age, gender, and race were adjusted. Model 3: age, gender, race, cancer, high blood pressure, use of hormone medication, take prescription for cholesterol, smoking, dietary inflammatory index, diabetes, PIR, triglycerides, fractured, and LDL-C were adjusted. *Abbreviation:PIR *Ratio of family income to poverty, *LDL-C *Low-density lipoprotein cholesterol

The relationship between WWI and BMD at all four sites was inconsistent across subgroups, as presented in Table 3. The association between WWI and BMD differed significantly across different genders at the total BMD site (*P* for interaction = 0.016), but did not show significant difference at the lumbar, pelvis, and femoral neck BMD sites (*P* for interaction > 0.05). The negative correlation between WWI and BMD was constant across different education levels at all sites (*P* for interaction > 0.05). In the subgroups of race/ethnicity, the associations between WWI and BMD did not show a statistically significant interaction at any of the sites (*P* for interaction > 0.05). The correlation varied in the subgroups of high blood pressure, with a significant interaction observed at the lumbar BMD site (*P* for interaction = 0.040) and total BMD site (*P* for interaction = 0.050), but no significant interaction at the pelvis and femoral neck BMD sites (*P* for interaction > 0.05). In the diabetes subgroups, the associations between WWI and BMD did not differ significantly at any of the sites (*P* for interaction > 0.05).

**Table 3:** Subgroup analysis of the association between weight-adjusted-waist index and bone mineral density

Subgroup	Lumbar BMD [β (95%CI)]	P for interaction	Pelvis BMD [β (95%CI)]	P for interaction	Femoral neck BMD [β (95%CI)]	P for interaction	Total BMD [β (95%CI)]	P for interaction
Sex		0.576		0.089		0.457		0.016
Male	-0.02 (-0.04, -0.00)		-0.06 (-0.08, -0.03)		-0.02 (-0.03, -0.01)		-0.03 (-0.05, -0.02)	
Female	-0.03 (-0.05, -0.01)		-0.03 (-0.05, -0.01)		0.00 (-0.02, 0.02)		-0.01 (-0.02, 0.01)	
Race/ethnicity		0.915		0.734		0.669		0.113
Non-Hispanic White	-0.03 (-0.05, -0.01)		-0.05 (-0.07, -0.02)		-0.01 (-0.03, 0.01)		-0.03 (-0.05, -0.01)	
Non-Hispanic Black	-0.02 (-0.05, 0.01)		-0.03 (-0.06, -0.00)		0.01 (-0.02, 0.03)		-0.00 (-0.02, 0.02)	
Mexican American	-0.04 (-0.08, 0.01)		-0.06 (-0.11, -0.01)		0.01 (-0.04, 0.06)		-0.03 (-0.06, 0.00)	
Other race	-0.02 (-0.06, 0.01)		-0.03 (-0.07, 0.00)		0.01 (-0.02, 0.05)		-0.02 (-0.04, 0.00)	
Education level		0.119		0.241		0.084		0.132
Less than high school	-0.05 (-0.06, -0.04)		-0.06 (-0.06, -0.05)		-0.05 (-0.06, -0.05)		-0.06 (-0.06, -0.05)	
High school	-0.04 (-0.04, -0.03)		-0.03 (-0.04, -0.03)		-0.05 (-0.05, -0.04)		-0.04 (-0.05, -0.04)	
More than high school	-0.04 (-0.05, -0.04)		-0.03 (-0.04, -0.03)		-0.05 (-0.05, -0.04)		-0.04 (-0.05, -0.04)	
High blood pressure		0.040		0.905		0.357		0.050
Yes	-0.05 (-0.07, -0.02)		-0.04 (-0.06, -0.02)		-0.01 (-0.04, 0.02)		-0.03 (-0.05, -0.02)	
No	-0.02 (-0.04, 0.00)		-0.04 (-0.06, -0.02)		0.00 (-0.01, 0.02)		-0.01 (-0.03, 0.00)	
Diabetes		0.295		0.210		0.480		0.092
Yes	-0.02 (-0.04, 0.01)		-0.06 (-0.08, -0.03)		-0.00 (-0.03, 0.02)		-0.02 (-0.04, 0.00)	
No	-0.03 (-0.05, -0.01)		-0.03 (-0.05, -0.01)		-0.00 (-0.02, 0.01)		-0.03 (-0.04, -0.01)	

Age, gender, race, cancer, high blood pressure, use of hormone medication, take prescription for cholesterol, smoking, dietary inflammatory index, diabetes, PIR, triglycerides, fractured, and LDL-C were adjusted. *Abbreviation:PIR *Ratio of family income to poverty, *LDL-C *Low-density lipoprotein cholesterol

## Discussion

In our cross-sectional study, which enrolled 40,568 eligible participants, we observed a negative association between WWI and BMD. Interestingly, there was a significant gender dependence on this association, indicating that a higher WWI may lead to a decrease in BMD among U.S. adults. These findings suggest that managing visceral fat distribution is important for bone metabolism.

This research is the first to examine the connection between BMD and WWI. Obesity and being overweight have always been seen as protective factors. BMI and BMD have a favorable correlation that has been shown in several research dating back at least 20 years [28, 29]. Researchers found that BMI, particularly in gender-specific populations and groups of menopausal women, decreased the incidence of bone loss and fractures [30, 31]. However, as a consequence of epidemiological research that refutes this idea, there has been a paradigm shift regarding obesity as a protective factor for osteoporotic fractures [32].

Numerous research has discovered nonlinear relationships and saturation effects between BMI and BMD in individuals of all ages, sexes, and ethnic backgrounds as a result of improvements in research methodology. These findings demonstrate that a simple linear positive correlation cannot adequately capture the relationship between BMI and BMD [8, 20, 33]. Additionally, research from various nations and locations has revealed considerable variations in BMI and fracture risk at various sites [34–36]. A prospective research of more than 800,000 middle-aged and older Spanish women, for instance, revealed that obesity reduced the risk of hip and pelvic fractures but raised the risk of proximal humeral fracture by 30% relative to the population at large [37].

However, the majority of studies investigating the relationship between obesity and BMD use the BMI and WC to assess obesity and are unable to distinguish between muscle mass, fat mass, and fat distribution. However, research examining bone metabolism must take body composition into account. According to Gnudi et al., there is only a connection between BMD and muscle mass in women who do not have osteoporosis, highlighting the significance of differentiating between various forms of body mass [38]. The WWI anthropometric measure is thought to be a marker of both high-fat mass and low-muscle mass [39]. There is a less substantial “obesity paradox” in WWI in the relationship between traditional indicators and metabolic diseases [40]. According to the most recent research, the obesity paradox may not actually exist, but rather, it is caused by the BMI’s inability to discern between muscle mass and fat mass [41]. Recent epidemiological research reveals that when assessing obesity and other illnesses, WWI performs better than several traditional indicators [13, 41–43].

According to the findings of the subgroup analysis, there were variations in the relationships between WWI and BMD at various sites depending on subgroups of gender, age, and race. The relationships between bone metabolism and several contributing variables vary by gender and race [26, 44]. For instance, a recent cross-sectional study indicated that non-Hispanic black women had a substantially stronger negative connection between metal exposure and BMD than did other groups [25]. Although it is believed that the relationship between WWI and abdominal fat and muscle mass is more sex- and race-specific [18, 45], the considerable age disparities in the NHANES for assessing BMD at various locations may be the cause of the subgroup discrepancies.

Uncertainty exists regarding the fundamental causes of this adverse relationship between BMD and WWI. Visceral fat has different metabolic properties from subcutaneous fat, and pro-inflammatory cytokines can speed up bone resorption and negatively affect BMD [46]. Additionally, there is strong proof that mesenchymal stromal/stem cells (MSC) have a negative correlation with osteoblast and adipocyte commitment. The systemic linkages between peripheral adipose depots and trabecular and cortical bone might be mediated by the same mechanisms that regulate MSC development locally within the marrow microenvironment [47, 48].

The adoption of a complicated multi-stage random sampling strategy and a high sample size are two of our study’s strengths, since they boost the reliability and representativeness of our findings [49, 50]. However, there are some limitations to our study that must be noted. For starters, because to the cross-sectional design, we were unable to establish a causal relationship between WWI and BMD. Due to database constraints, we were unable to incorporate data on all factors that influence bone metabolism, such as menopause and medication usage. This was done in order to keep the sample size large enough. Despite these restrictions, the present association between WWI and BMD was steady enough that it was less likely to be considerably impacted by characteristics that were not included.

## Conclusion

Our study provides new evidence for a complex link between obesity and bone metabolism, as we found a significant and negative association between WWI and BMD in US adults. This highlights the importance of managing visceral fat distribution in bone metabolism and sheds light on the limitations of using traditional obesity measurements, such as BMI and WC, to assess bone health.

### Supplementary Information

**Additional file 1.**

## Data Availability

The survey data are publicly available on the internet for data users and researchers throughout the world ( www.cdc.gov/nchs/nhanes/ ).

## Abbreviations

WWI: Weight-adjusted waist circumference index
BMD: Bone mineral density
BMI: Body mass index
WC: Waist circumference
NHANES: National Health and Nutrition Examination Survey
NCHS: National Center for Health Statistics
LDL-C: Low-density lipoprotein cholesterol
PIR: Income-to-Poverty Ratio
MSC: Mesenchymal stromal/stem cells
RCS: Restricted cubic spline
