# Supplementary material for: Associations between weight-adjusted waist index and bone mineral density: results of a nationwide survey
Source: BMC Endocr Disord. 2023 Aug 3;23:162. doi: 10.1186/s12902-023-01418-y (PMC10399051; doi:10.1186/s12902-023-01418-y)
Supplement: Supplementary file 1 — Additional file 1. [file 12902_2023_1418_MOESM1_ESM.docx]

Supplementary file: Methods of obtaining the main variables and the use of weights

To obtain bone mineral density (BMD) data using the NHANES database, we followed the steps below:

File selection: In the NHANES database, for the BMD data, we used the "Dual-Energy X-ray Absorptiometry - Whole Body (DXX_)" file and the "Dual-Energy X-ray Absorptiometry - Femur (DXXFEM_)" file. Among them, lumbar BMD, pelvis BMD, and total BMD are extracted from Dual-Energy X-ray Absorptiometry - Whole Body (DXX_), and femoral BMD is extracted from Dual-Energy X-ray Absorptiometry - Femur (DXXFEM_).

As for the Dietary Inflammation Index (DII), it is a novel tool that assesses an individual's diet on a continuum from maximally anti-inflammatory to maximally pro-inflammatory. The DII is calculated from 24-hour dietary reviews and dietary intake data from the Nutrient Database, including micronutrients, macronutrients, and other food components. The detailed calculation method is as follows:

The dietary database's nutrient information of the first day was selected as the accurate nutrient intake for each participant in the study. A total of 27 nutrients, including total energy, fat, saturated fat, monounsaturated fat, polyunsaturated fat, ALA (oct adecatrienoic acid), EPA (eicosapentaenoic acid), DHA (docosahexaenoic acid), docosapentaenoic acid, linoleic acid (octadecadienoic acid), arachidonic acid (eicosatetraenoic acid), protein, carbohydrates, fiber, alcohol, cholesterol, niacin , vitamins (A, B1, B2, B6 , B12, C, D, E), iron, zinc, selenium, magnesium, folic acid, beta carotene, and caffeine were considered in the calculation of the DII. The n-3 fatty acids were equal to the sum of ALA, EPA , DHA, and docosapentaenoic acid, and the n-6 fatty acids were equal to the sum of linoleic acid and arachidonic acid.

The average and variability of each nutrient were obtained from the World Diet Standards Library and used to transform the Z-scores of the respective nutrient into Z-transformed scores. The Z-transform fraction of each nutrient was then transformed into percentiles, and the resulting distribution for each nutrient level was made symmetrical around 0 (zero) by doubling the transformed percentiles and subtracting 1. The bounds of this distribution are −1 (maximum anti-inflammatory) and +1 (maximum pro-inflammatory). The final DII score is obtained by multiplying each nutrient level by its respective corresponding inflammatory fraction and summing the result.

For the NHANES weights the following specific methods were used:

(i) All variables were collected in an in-home interview and weights used wtint2yr;

Only if all variables of the study were collected in the in-home interview, weights were used wtint2yr, if there were other variables collected in other ways, proceed to the next section;

(ii) Some variables were collected as MEC, weights were used wtmec2yr;

If all variables are only collected in two ways, in-home interview and MEC check, use wtmec2yr for weights; if there are also sub-sample variables (no 24-hour dietary recall variables), refer to point (iii) to select weights; if 24-hour dietary recall variables are included then refer directly to point (iv) to select weights;

(iii) If some of the variables are part of the survey subsample, the corresponding subsample weights are used;

If the variables studied included all three weights, wtint2yr, wtmec2yr and the self-sample weight, the appropriate subsample weight was selected; because all participants were interviewed (in-home interview) (population 1), some of those interviewed were examined for MEC (population 2), and of those examined for MEC, only those who fasted for 8 (population 3: subsample population), i.e. population 3 (subsample population) < population 2 < population 1, according to the core principle of weight selection, so the weight corresponding to the subsample variables was chosen as the final weight.

(iv) Some variables from 24-hour dietary recall

1. hour dietary recall is not a sub-sample variable, but participants who completed this part of the survey were weighted in a particular way, as there may be differences in dietary intake between weekdays and weekends during the week, and this weight adjusts for these differences. Whenever variables in the study were collected as 24-hour dietaryrecall (whether or not wtint2yr, wtmec2yr or subsample weights were included), the final weight was wtdrd1 (day 1)/wtdrd2 (2 days).
